# Multilevel Analysis of the Patterns of Physical-Mental Multimorbidity in General Population of São Paulo Metropolitan Area, Brazil

**DOI:** 10.1038/s41598-019-39326-8

**Published:** 2019-02-20

**Authors:** Yuan-Pang Wang, Bruno P. Nunes, Bruno M. Coêlho, Geilson L. Santana, Carla F. do Nascimento, Maria Carmen Viana, Isabela M. Benseñor, Laura H. Andrade, Alexandre D. P. Chiavegatto Filho

**Affiliations:** 1 Nucleo de Epidemiologia Psiquiatrica (LIM-23), Instituto de Psiquiatria, Hospital das Clinicas HC FMUSP, Faculdade de Medicina, Universidade de Sao Paulo, Sao Paulo, SP Brazil; 2 Nursing Department, Federal University of, Pelotas, RS Brazil; 3 Department of Epidemiology, School of Public Health, University of São Paulo, Sao Paulo, SP Brazil; 4 Department of Social Medicine, Federal University of Espírito Santo, Vitória, ES Brazil; 5 Center for Clinical and Epidemiological Research, Hospital Universitario HU USP, Universidade de Sao Paulo, Sao Paulo, SP Brazil

## Abstract

Chronic diseases are often comorbid and present a weighty burden for communities in the 21^st^ century. The present investigation depicted patterns of multimorbidity in the general population and examined its association with the individual- and area-level factors in an urban sample of non-elderly adults of Brazil. Data were from the cross-sectional São Paulo Megacity Mental Health Survey, a stratified multistage area probability sampling investigation. Trained interviewers assessed mental morbidities and asked about physical conditions for 1,571 community-dwelling women and 1,142 men, aged between 18 and 64 years. Principal component analysis depicted patterns of physical-mental multimorbidity, by sex. Following, the patterns of multimorbidity were subjected to multilevel regression analysis, taking into account individual- and area-level variables. Three patterns of clustering were found for women: ‘irritable mood and headache’, ‘chronic diseases and pain’, and ‘substance use disorders’. Among men, the patterns were: ‘chronic pain and respiratory disease’, ‘psychiatric disorders’, and ‘chronic diseases’. Multilevel analyses showed associations between multimorbidity patterns and both individual- and area-level determinants. Our findings call for a reformulation of health-care systems worldwide, especially in low-resource countries. Replacing the single-disease framework by multi-disease patterns in health-care settings can improve the ability of general practitioners in the health-care of person-centred needs.

## Introduction

Multimorbidity is a taxing concept to delimitate^1^, but it can be defined as a non-random association pattern between diseases^2^. Generally, multimorbidity is conceptualized as the co-occurrence of multiple chronic or long-term medical conditions. Multimorbidity includes both physical and mental illnesses and is distinguished from comorbidity due to the absence of an index disease or condition as is the case of the second^3^. The tendency of chronic conditions to cluster into distinct configurations represents the norm in older age people, resulting in higher disease persistence, functional disability, polypharmacy, health-care service use, and mortality^3–5^.

Although multimorbidity is a worldwide concern, most of beyond chance combination of medical and psychiatric conditions has been recognized in developed countries^6^. However, this phenomenon is not restricted to the elderly living in high-income countries (HICs). Multimorbidity affects more young people in low- and middle-income countries (LMICs) than in HICs^7^. Chronic non-communicable diseases represent a large share of disease burden in LMICs^8^, which start during peak economically active years of age. For example, the World Health Survey has estimated a mean prevalence of multimorbidity of 7.8% in 28 LMICs^9^. Moreover, in WHO’s Study on global AGEing and adult health (SAGE), over one-fifth of participants from six LMICs reported multiple morbidities^10^. All this burden of chronic multimorbidity contradicts the preceding belief that mortality-related burden still prevails in LMICs. The complex service needs of growing multimorbid populations in low-resource countries challenge policymakers to restructure health-care delivery.

In the general population, episodes of ill-health seem unevenly distributed among individuals, wherein some groups experience more diseases than others^11^. When pairs of conditions co-occur more frequently than expected, putative shared risk factors might be involved or one of the two processes operates as the risk factor for the other^5^. This morbidity-dependent mechanism might rely on a self-perpetuating and mutually reinforcing causality^1^. The reciprocal influence of general medical conditions and mental disorders is apparent when individuals reporting long-lasting psychiatric disorders also present more medical illnesses^12^. For example, generalized anxiety and dysthymia are the strongest predictors of medical multimorbidity. Similarly, hypertension and asthma are the strongest predictors for psychiatric multimorbidity. In this sense, the extent of multimorbidity has also been associated with age, sex, educational level, country’s income, and social inequality^6,13,14^. As a consequence, the resulting health-care needs of multimorbid people suggest a pressing reorganization of the health system in forthcoming decades^15^. Considering insufficient budget allocated to health in LMICs, reorienting health-care resources to identify and target adverse effects of morbidity burden is the utmost priority.

Over and beyond individual predictors, area-level or contextual factors may exert a joint effect^16,17^. Across all ages, individuals from deprived areas are more likely to present multimorbidity than those living in affluent areas^13,14^. Nevertheless, the majority of studies examining patterns of multimorbidity and area-level determinants were performed with clinical samples in developed countries, where social inequality is deemed to be lower than in LMICs^6,17^.

The numerous service needs of growing multimorbid populations challenge policymakers to rethink health-care delivery, but comprehensive information of health-care utilization by subjects with multimorbidity is scant, in terms of provision and expenditure^16,18^. Furthermore, considerable gaps still remain in the literature: Which are the individual- and area-level determinants of multimorbidity in the general population of LMICs? What is its impact on health-care systems?

It was estimated that at least 19 million adults present multimorbidity in Brazil^19^. A recent publication on global burden of diseases of Brazil has demonstrated that the morbidity profile has not changed substantially in the country since 1990: most of the leading causes of years lived with disability (YLDs) are non-communicable and chronic diseases (e.g., low back & neck pain, migraine, depressive disorders, anxiety disorders, etc.)^20^. In past decades, despite a gradual improvement, with the expansion of universal health coverage, structural problems still persist in health-care organisation and governance in the Brazilian health system. Moreover, limited funding and suboptimal resource allocation lead to persistent regional disparities in access to health-care services and health indicators^21,22^. For dwellers of an urbanized São Paulo metropolitan area, the 4^th^ densely populated area in the globe, the availability of health-care services remains below standards of adequacy^23–25^. In the current study, we aim to investigate patterns of multimorbidity in the non-elderly general population of the metropolitan area of São Paulo^26^, located in a middle-income country. We also examine the association of multimorbidity with health-care utilization, as well as the determinants of individual- and area-level variables.

## Methods

This is a multilevel analysis of the influence of individual- and area-level factors on patterns of physical–mental multimorbidity and health-care use in the general population of the metropolitan area of São Paulo.

### Sampling

Cross-sectional data were drawn from the São Paulo Megacity Mental Health Survey^26^, the Brazilian branch of the World Mental Health Survey initiative^27^. A household representative sample of individuals aged 18 years or older living in the São Paulo metropolitan area was selected through stratified, multistage area probability sampling. This area comprises the city of São Paulo and its 38 surrounding municipalities, with an estimated population of 21 million inhabitants^28^. At the time of data collection, from May 2005 to May 2007, there were approximately 11 million inhabitants aged 18 years or older. After exclusion of 200 elderly with cognitive impairment, a total of 5,037 eligible respondents accepted to be interviewed in their households. The global response rate was 81.3%. A detailed description of the sampling procedure was presented elsewhere^29^.

**Figure 1:**
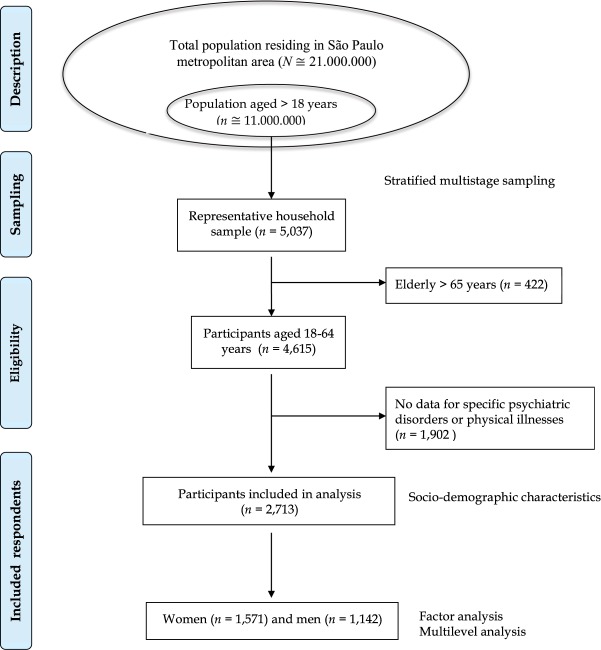
Flow diagram of the study sampling.

In the current analysis, to avoid the cumulative aging effect of multiple diseases, we excluded 422 participants aged over 65 years, resulting in a representative sample of 4,615 subjects aged between 18 and 64 years. Specific psychiatric disorders (e.g., post-traumatic stress, obsessive-compulsive, and premenstrual dysphoric disorders) were asked only for a representative, probability sub-sample. We excluded 1,902 individuals because they were not assessed for all morbidities. Therefore, data from 2,713 respondents whose answers contained complete dataset on psychiatric and physical morbidity were analysed (Fig. 1).

For multilevel analysis, characteristics of 69 areas of respondents’ residence were considered. The area-level of the model took into account the 31 administrative regions or “*sub-prefeituras*” of the city of São Paulo (average of 355,467 residents) and each one of the 38 municipalities located in this metropolitan area (average of 232,751 residents).

### Assessment of socio-demographics

Information on sex, age, marital status, education, family income, and employment were collected. Sex was coded as male or female. Age was categorized as 18–34, 35–49, and 50–64-year-old (yo) brackets. Marital status was defined as married/cohabitating, previously married (widowed/separated/divorced) and never married. Years of schooling were coded as fundamental (≤4 years), basic (5–8 years), high school (9–11 years) and college (≥12 years). Socioeconomic status (SES) was expressed as the family income divided by the average income of the sample, resulting in four quartiles. Employment status was categorized as employed, student, homemaker, retired and unemployed.

### Assessment of mental disorders

The World Mental Health version of the Composite International Diagnostic Interview (WMH-CIDI) was used. The WMH-CIDI is a fully structured instrument applied by lay evaluators^30^, which has acceptable validity and reliability^31^. The algorithm of this instrument allows generating the diagnosis of mental disorders according to DSM-IV and ICD-10.

Four classes of 12-month mental disorders (anxiety, mood, impulse-control, and substance use disorders [SUD]), plus premenstrual dysphoria (in women) and heavy drinking, were considered. Heavy drinking was defined as the consumption of more than 40 g/day of alcohol for men and more than 20 g/day for women^32^.

Anxiety disorders comprised panic, agoraphobia without panic, simple phobia, social phobia, generalized anxiety, adult separation anxiety, obsessive-compulsive, and post-traumatic stress disorders. Mood disorders included major depression, dysthymia, and bipolar disorder. Impulse-control disorders were intermittent explosive, oppositional-defiant, conduct, and attention-deficit/hyperactivity disorder. The substance use disorders (SUD) covered alcohol and drug abuse and dependence.

### Assessment of physical illnesses

Chronic physical illnesses were assessed with a standard checklist of diseases. This checklist, used in all studies of the World Mental Health Initiative^27^, has produced more adequate illness prevalence than those estimates resultant from inventories with open-ended questions^33,34^. In earlier studies^35,36^, it has also demonstrated a moderate to good concordance with clinical records. Respondents reported whether a doctor or other health professional ever told them they had the condition in the previous year.

Ten prevalent medical conditions were considered in the analysis: cardiovascular diseases (heart attack, heart disease, and stroke), hypertension, diabetes mellitus, arthritis, chronic musculoskeletal pain, headache or migraine, digestive, respiratory (seasonal allergies, asthma, obstructive pulmonary disease, and emphysema), neurological diseases (Parkinson disease, epilepsy, and multiple sclerosis), and cancer.

### Severity assessment

Respondents self-rated the impairment caused by each morbidity assessed during the worst month in the past year using the Sheehan Disability Scale^37^. The 10-point visual analogue scale assessed disability in the following domains: work role performance, household maintenance, social life, and intimate relationship. The scale scores ranged from none, 0; mild, 1–3; moderate, 4–6; to severe, 7–10. Respondents who had reported more than 1 disorder were assigned to the highest score for any single disorder.

Participant’s functional status was categorized as severe if the respondent has shown a high level of impairment on the Sheehan Disability Scale in at least one of the morbidities presented; were diagnosed with bipolar disorder, or substance dependence with physiologic signs; had attempted suicide in the past year^38^, in conjunction with one or more core DSM-IV psychiatric disorder.

Among those cases that were not categorized as severe, respondents were labelled ‘moderate’ if they had at least one disorder with a moderate level of impairment on any domain or substance dependence without physiological signs. The remaining respondents with any active disorder were categorized as ‘mild’. Accordingly, 12-month disease severity level of each respondent was classified as ‘no’, ‘mild’, ‘moderate’, or ‘severe’.

### Use of service

The use of any medical service was asked through the question: “*Do you have a physician who you usually consult when you need routine care*?”. The availability of a regular physician for routine medical treatment in the past 12 months was analysed as absence (0) or presence (≥1) of treatment access.

Respondents were also asked whether they “*ever saw any professional treatment for problems with their emotions*, *nerves*, *mental health*, *or use of substances*” in the 12 months prior to the interview. In the present paper, the use of mental health-care refers to either a general medical or mental health sector for the treatment of any mental disorders. The general medical sector included primary care physicians, nurses, or other health-care professionals. The specialized mental health sector included psychiatrists and other professionals (psychologists, social workers, or counsellors in a mental health setting).

### Statistical analysis

In the analytic approach, the variance estimation procedures with complex sample survey data were employed to account the stratified multistage area sampling design. To adjust for differences in the chance of selection and non-response within-households, weights were applied to adjust the prevalence rates. The differential sampling of WMH-CIDI was corrected, wherein a sub-sample of participants has answered to all questions about psychiatric and physical morbidities. Finally, a post-stratification weight was used to equate the sample distribution to the population distribution in the 2000 census^28^.

First, the weighted prevalence of each disease variable was calculated both for the total sample and by sex. Between-sex differences were assessed by chi-square test. Likewise, we determined the number of diseases in the same respondent, both for the total sample and by sex. As a substantial difference was apparent, we built a sex-specific histogram for the number of diseases by each age bracket. Also, selected prevalent conditions (over 5%) were analysed through a graphical matrix in accordance with family socioeconomic status (SES) quartiles. The high-SES quartile was compared to low-SES quartile in a bubble graph. Accordingly, this graph of conditional probabilities on the pairwise correlation between selected prevalent conditions further provides a visual appraisal of these relationships. The conditional relationship of multimorbidity was shown as the percentage of persons who presented both conditions.

Second, since all observed variables on psychiatric and physical morbidities were binary, a principal component analysis determined the covariance structure of the data and built the factor scores of each extracted component, separately by sex. Both Kaiser’s eigenvalue > 1.0 rule-of-thumb criteria^39^ and Cattell’s scree test^40^ established the number of components to extract, and both orthogonal and oblique rotations were applied for interpretation.

After the identification of models, we investigated the relationship between each multimorbidity pattern and risk factors, i.e., between latent and observed variables. Five multilevel regression models estimated the simultaneous influence of participants’ individual- and area-level characteristics on each sex-specific pattern of multimorbidity^41^. Individual-level information was included in the first model: socio-demographic variables (age bracket, marital status, and years of education), morbidity severity, and access to health-care facilities (medical treatment and mental health-care service).

Thereafter, four area-level models were sequentially incorporated to the equation for examining the influence of following area-level administrative information: education, median income, socio-economic inequality (Gini coefficient), and violence (homicide rate per 100.000 inhabitants). Area-level median income and education (measured by the proportion of individuals that completed basic education) were categorized as low, medium and high by tertiles to allow for possible non-linearity, using the results from the 2010 Census^28^.

The intraclass correlation coefficient (ICC) was estimated for each random intercept model to determine the proportion of total variance explained by area-level variance while accounting for the non-independence of individual observations within groups. In other words, ICC expresses the correlation between individuals belonging to the same group. For interpretation, a non-zero ICC implies that the observations are not independent. The lower the ICC between two observations within the same cluster, the lower the variability is between the clusters and the higher the variability is within the clusters. If all the responses from observations in the same cluster are exactly the same, the ICC equals 1. If all the observations are independent of one another, the ICC equals zero.

Descriptive and principal component analyses were performed using the SPSS/PASW 17 software (www-01.ibm.com) and the multilevel models were estimated with R 3.2.2 software (www.cran.r-project.org). The level of significance was set at *p* < 0.05 for two-tailed tests.

### Ethics approval and informed consent

The Research and Ethics Committee of the University of São Paulo Medical School approved the procedures for recruitment, obtaining informed consent, and protecting human subjects during field works. Respondents were interviewed after signing an informed written consent and being assured of confidentiality.

The authors assert that all procedures contributing to this work comply with the ethical standards of the relevant national and institutional committees on human experimentation and with the Helsinki Declaration of 1975, as revised in 2008 and, also, the authors assert that all procedures contributing to this work comply with the ethical standards of the relevant national and institutional guides.

## Results

### Socio-demographic characteristics of the sample

Table 1 depicts socio-demographic characteristics of the 2,713 community-dwelling respondents aged 18–64 years old (yo) in the São Paulo metropolitan area. Women represented 52.4% of the sample, around half of the respondents (48.1%) were aged 18–34 yo, 35.5% were 35–49 yo and 16.4% were 50–64 yo. The majority (61.2%) had average schooling of 5–11 years and 60.9% was married or cohabiting. Family income, or the proxy for socio-economic status (SES), was evenly distributed across groups: low (22.6%), middle-low (28.5%), medium-high (22.7%), and high (26.2%). The majority (68.9%) was employed at the time of the interview.

**Table 1:** Descriptive characteristics of the participants of the São Paulo Megacity Mental Health Survey (*N* = 2,713, aged 18–64 years).

Variable	*n*	%
**Sex**		
Men	1,142	47.63
Women	1,571	52.37
**Age**, **years**		
18–34 yo	1,091	48.11
35–49 yo	1,028	35.45
50–64 yo	594	16.44
**Education (years of schooling)**		
Low (0–4)	644	19.34
Low-average (5–8)	700	22.82
High-average (9–11)	952	38.35
High (≥12)	417	19.49
**Marital status**		
Married/cohabiting	1,734	60.87
Previous married	492	13.60
Never married	487	25.53
**Family income**		
Low	694	22.58
Low-average	759	28.47
High-average	615	22.72
High	645	26.23
**Employment status**		
Employed	1,706	68.94
Student	30	1.71
Homemaker	453	12.98
Retired	140	3.94
Unemployed	384	12.43

% Weighted proportion.

### Prevalence of 12-month morbidities

In the total sample, anxiety (20.7%) and mood disorders (12.5%) were the most prevalent psychiatric disorders in the past year (Table 2). For the female sub-sample, anxiety and mood disorders were also prevalent, respectively in 27.2% and 17.5% of women. Premenstrual dysphoria was reported by more than half of women (51.2%). In the male sub-sample, the most prevalent disorders were heavy drinking (16.6%) and anxiety disorders (13.6%). Significant sex differences were observed for all classes of psychiatric disorders, except impulse-control disorders.

**Table 2:** Prevalence of 12-month mental disorders and physical illnesses in the general population of the São Paulo metropolitan area, total sample (*N* = 2,713, aged 18–64 years) and by sex.

	Total (*n* = 2,713)			Women (*n* = 1,571)			Men (*n* = 1,142)			χ^2^	p-value
	*n*	%	SE	*n*	%	SE	*n*	%	SE		
**Psychiatric disorders**											
Anxiety disorder	795	20.7	0.9	553	27.2	1.6	242	13.6	1.1	38.8	< 0.0001
Mood disorder	548	12.5	0.9	410	17.5	1.5	138	7.1	0.8	42.6	* < *0.0001
Heavy drinking	278	10.3	0.8	88	4.7	0.6	190	16.6	1.6	68.9	* < *0.0001
Impulse-control disorder	189	4.8	0.5	103	4.6	0.6	86	5.0	0.7	0.3	0.59
Substance use disorder	161	4.1	0.4	37	1.8	0.3	124	6.7	0.8	46.6	< 0.0001
Premenstrual dysphoria^c^	—	—	—	843	51.2	2.3	—	—	—	NA	—
**Physical illnesses**											
Musculoskeletal pain	1,083	32.8	1.5	700	37.4	2.2	383	27.7	1.7	15.8	0.0002
Head/migraine	984	30.7	1.5	736	40.9	2.2	248	19.5	2.1	44.5	< 0.0001
Respiratory disease	705	24.7	1.3	496	30.1	1.8	209	18.7	2.0	14.0	0.0005
Hypertension	579	17.1	1.1	359	18.0	1.4	220	16.2	1.7	0.6	0.45
Arthritis	244	6.5	0.7	195	9.3	1.1	49	3.5	0.8	17.5	0.0001
Cardiovascular disease	196	4.3	0.4	124	4.7	0.6	72	3.7	0.6	1.1	0.30
Diabetes	147	3.9	0.5	88	3.7	0.6	59	4.0	0.8	0.1	0.79
Digestive disease	118	2.2	0.3	78	2.6	0.4	40	1.7	0.3	2.8	0.10
Neurological disease	66	1.6	0.3	37	1.6	0.3	29	1.5	0.4	0.1	0.78
Cancer	19	0.5	0.1	14	0.6	0.2	5	0.4	0.2	0.4	0.51

*n*: subsample of respondents aged between 18–64 years, considering those who have answered to all mental disorders and general medical conditions.

%: weighted prevalence.

^c^Premenstrual dysphoria refers to women.

SE: standard error; NA: not applicable.

For physical illnesses, musculoskeletal pain (32.8%), headache/migraine (30.7%), respiratory disease (24.7%), and hypertension (17.1%) were the most prevalent conditions in the total sample (Table 2). Women more likely reported painful conditions (musculoskeletal pain, headache/migraine, and arthritis) and respiratory diseases than men.

### Number and type of multimorbidity

**Figure 2:**
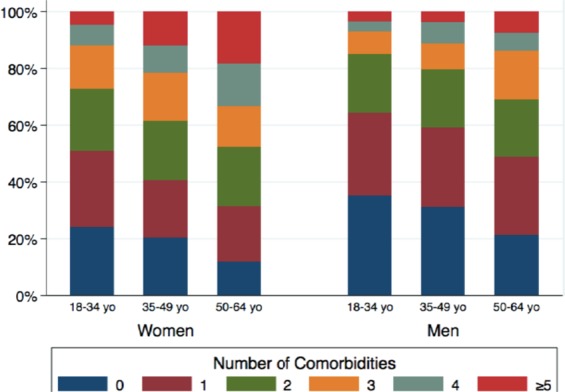
Number of comorbid conditions by sex and age bracket.

Multimorbidity was common for people younger than 65 years and the prevalence increased with age. Almost half of the respondents reported more than one condition during the past year, being 20.7% reported two and the remaining 27.8%, three to 10 co-occurring conditions (Table 3). Women significantly complained of more diseases than men, as well as more multimorbidity. Figure 2 shows the number of comorbid conditions by sex and age bracket. A salient dose-response gradient between age and number of morbidities was observed for both sexes: the higher the age bracket, the greater the proportion of concomitant conditions. In general, women reported more comorbid conditions than men for all age brackets.

**Table 3:** Total number and proportion of morbidities in the general population of the São Paulo metropolitan area, total sample (*N* = 2,713) and by sex.

Number of conditions	Total		Women^†^		Men		p
	*n*	%	*n*	%	*n*	%	
0	456	24.43	191	19.19	265	30.19	<0.0001
1	616	27.10	308	24.44	308	30.02	0.0620
2	577	20.68	334	21.66	243	19.60	0.3800
3	440	12.90	292	14.74	148	10.88	0.0284
4	294	7.92	200	10.14	94	5.48	0.0015
5	184	4.09	134	5.79	50	2.21	0.0003
6	75	1.68	56	2.43	19	0.86	0.0052
7	43	0.75	36	1.12	7	0.34	0.0201
8	21	0.33	16	0.40	5	0.27	0.4938
9	6	0.11	3	0.07	3	0.15	0.4534
10	1	0.01	1	0.02	—	—	NA
**Total**	**2,713**	**100.00**	**1,571**	**100.00**	**1,142**	**100.00**	

^**†**^Includes premenstrual dysphoric disorder.

**Figure 3:**
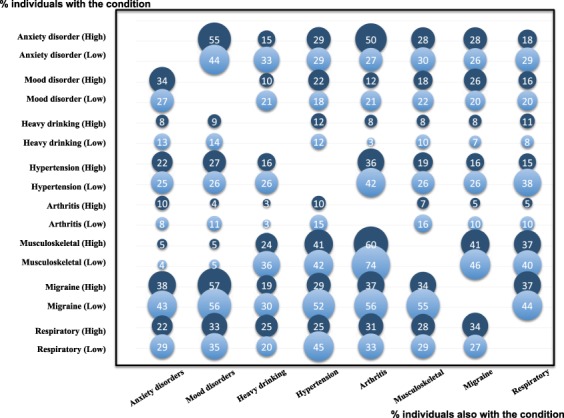
Conditional pairwise proportion of selected multimorbidities among people with common and frequent morbidities from the most affluent (dark blue) and the most deprived (light blue) socioeconomic quartiles.

Regarding the co-occurrence of different conditions, the most common pairwise relationship was between musculoskeletal pain and arthritis, anxiety and mood disorders, and mood disorders and migraine. When respondents from the higher and the lower SES were compared, substantial influence of the socio-economic dissimilarity was demonstrated (Fig. 3). For example, when high-SES respondents with anxiety were compared with those low-SES ones, the proportion of arthritis was twice higher among the former. Conversely, the multimorbidity of heavy drinking and anxiety was doubled among respondents from low SES.

### Patterns of multimorbidity

For both women and men, the model selected by the Kaiser’s criterion was the one with 3 factors and explained 31.2% and 30.4% of the total variance, respectively (Table 4). Nevertheless, the morbidity composition and factor loading in each factor varied by sex.

**Table 4:** Pattern matrix of 12-month multimorbidity of psychiatric disorders and general medical conditions in the general population of the São Paulo metropolitan area (*N* = 2,713, aged 18–64 years), by sex.

Variable	Women				Men			
	Factor 1 Irritable mood & headache	Factor 2 Chronic diseases & chronic pain	Factor 3 Substance use disorders	*h* ^2^	Factor 1 Chronic pain & respiratory disease	Factor 2 Psychiatric disorders	Factor 3 Chronic diseases	*h* ^2^
Premenstrual dysphoria	**0**.**66**	0.16	0.06	0.43				
Mood disorder	**0**.**64**	0.02	0.05	0.41	0.24	**0**.**57**	0.20	0.46
Anxiety disorder	**0**.**61**	−0.04	0.08	0.38	0.14	**0**.**47**	0.29	0.34
Impulse-control disorder	**0**.**47**	0.12	0.23	0.26	−0.09	**0**.**66**	0.06	0.44
Headache/migraine	**0**.**43**	−0.14	−0.09	0.24	**0**.**68**	0.03	−0.06	0.46
Respiratory disease	0.26	−0.03	−0.09	0.09	**0**.**38**	0.00	0.01	0.14
Hypertension	−0.08	**−0**.**72**	0.10	0.49	0.01	−0.02	**0**.**69**	0.50
Cardiovascular disease	0.02	**−0**.**60**	0.07	0.35	0.04	0.02	**0**.**55**	0.31
Diabetes mellitus	−0.15	**−0**.**59**	0.11	0.34	−0.26	0.01	**0**.**68**	0.48
Arthritis	0.05	**−0**.**54**	−0.11	0.34	**0**.**35**	−0.17	0.22	0.21
Musculoskeletal pain	0.34	**−0**.**37**	−0.16	0.35	**0**.**63**	0.08	0.09	0.43
Digestive disease	0.21	−0.25	−0.10	0.14	**0**.**34**	−0.02	0.02	0.12
Heavy drinking	0.14	−0.12	**0**.**76**	0.57	−0.11	**0**.**39**	−0.18	0.20
Substance use disorder	0.21	−0.07	**0**.**69**	0.49	−0.06	**0**.**61**	−0.10	0.38
Neurological disease	0.18	−0.03	−0.21	0.09	0.19	0.18	−0.01	0.07
Cancer	0.05	−0.01	−0.20	0.04	0.12	−0.01	−0.07	0.02
% of variance explained	13.7	10.1	7.4		13.1	9.6	7.7	
**Between-factor correlation**								
Factor 1	1.00				1.00			
Factor 2	−0.17	1.00			0.08	1.00		
Factor 3	−0.09	0.16	1.00		0.13	−0.01	1.00	

*h*^2^: communality.

Among women, the first component was labelled as “irritable mood and headache” and encompassed premenstrual dysphoric, mood, anxiety, impulse-control disorders, and headache/migraine. The second component of “chronic diseases and pain” included hypertension, cardiovascular illnesses, arthritis, diabetes, and musculoskeletal pain. The third component, “substance use”, included heavy drinking and substance use disorders (SUD). Respiratory, digestive, neurological illnesses and cancer presented low communality (<0.15) and did not contribute to the model (factor loading < 0.3).

For men, the first component, “chronic pain and respiratory disease”, included headache/migraine, musculoskeletal pain, arthritis, respiratory, and digestive illnesses. The second component was named “psychiatric disorders” and included impulse-control, mood, anxiety disorders, SUD, and heavy drinking. Finally, the third component described a dimension of “chronic diseases” that contained hypertension, cardiovascular illnesses, and diabetes. Neurological illness and cancer did not contribute to the model.

### Multilevel analysis

Multilevel analysis, or hierarchical models, is an analytical approach that permits the examination of simultaneous effects of individual-, and group-level variables on outcome variables, allowing depicting the isolated and combined influence of variables of each level on the outcome variable^41^. Therefore, ten sex-specific linear models examined the influence of individual- and contextual-level variables, separately for women and men. In general, the intraclass correlation (ICC) varied greatly across models (Supplementary Table S1). Table 5 shows the model of each sex-specific pattern of multimorbidity that showed the lowest ICC. Individual-level variables like age, education, Sheehan category of disease severity, and use of health-care services were associated with increased likelihood of presenting multimorbid patterns. Jointly, the area-level variables explained 24% and 32% of the variance, respectively in women with “irritable mood” and men with “psychiatric disorders”. The remaining models explained less than 20% of the variance, and no influence of area-level variables was shown among men with “chronic diseases” (ICC = 0).

**Table 5:** Multilevel analysis of patterns of multimorbidity in the general population of the São Paulo metropolitan area, by sex.

	Women						Men					
	Irritable mood & headache		Chronic diseases & pain		Substance use disorders		Chronic pain & respiratory disease		Psychiatric disorders		Chronic diseases	
	β	(95% CI)	β	(95% CI)	β	(95% CI)	β	(95% CI)	β	(95% CI)	β	(95% CI)
**Age bracket**												
18–34 yo^†^												
35–49 yo	0.04	(−0.02; 0.10)	−0.31*	(−0.40; −0.23)	−0.11*	(−0.21; −0.02)	0.04	(−0.06; 0.15)	−0.18*	(−0.25; −0.11)	0.30*	(0.20; 0.41)
50–64 yo	−0.07	(−0.15; 0.00)	−0.99*	(−1.10; −0.89)	−0.30*	(−0.42; −0.18)	0.08	(−0.05; 0.21)	−0.30*	(−0.38; −0.22)	0.83*	(0.71; 0.95)
**Marital status**												
Single^†^												
Married/Cohabiting	0.02	(−0.05; 0.10)	−0.03	(−0.13; 0.07)	−0.05	(−0.17; 0.06)	0.19*	(0.07; 0.31)	0.00	(−0.08; 0.07)	0.05	(−0.06; 0.16)
Separated/Widowed/Divorced	0.00	(−0.09; 0.09)	0.06	(−0.06; 0.18)	0.06	(−0.07; 0.20)	−0.04	(−0.22; 0.14)	0.02	(−0.09; 0.13)	0.10	(−0.07; 0.26)
**Educational level**												
1^†^												
2	0.04	(−0.03; 0.11)	0.21*	(0.11; 0.31)	−0.07	(−0.18; 0.05)	−0.08	(−0.21; 0.04)	0.02	(−0.06; 0.10)	−0.05	(−0.17; 0.06)
3	0.01	(−0.06; 0.09)	0.30*	(0.20; 0.40)	0.01	(−0.10; 0.13)	−0.23*	(−0.35; −0.10)	0.01	(−0.07; 0.09)	−0.09	(−0.20; 0.03)
4	−0.02	(−0.11; 0.07)	0.50*	(0.38; 0.63)	0.02	(−0.12; 0.16)	−0.16*	(−0.31; −0.01)	0.00	(−0.10; 0.09)	−0.13	(−0.27; 0.00)
**Severity**												
1	1.82*	(1.73; 1.90)	−0.28*	(−0.39; −0.16)	0.42*	(0.29; 0.55)	0.66*	(0.49; 0.83)	2.37*	(2.26; 2.48)	0.36*	(0.20; 0.52)
2	1.45*	(1.37; 1.54)	−0.13*	(−0.24; −0.01)	0.04	(−0.09; 0.16)	0.59*	(0.41; 0.77)	1.67*	(1.56; 1.79)	0.75*	(0.58; 0.92)
3	1.23*	(1.14; 1.32)	−0.09	(−0.21; 0.04)	0.23*	(0.09; 0.37)	−0.01	(−0.17; 0.16)	1.57*	(1.47; 1.67)	0.30*	(0.15; 0.45)
4^†^												
**Medical treatment**												
No^†^												
Yes	0.36*	(0.28; 0.44)	−0.21*	(−0.32; −0.10)	−0.22*	(−0.34; −0.09)	0.25*	(0.06; 0.43)	0.08	(−0.04; 0.20)	0.33*	(0.16; 0.50)
**Mental health-care**												
No^†^												
Yes	−0.13*	(−0.21; −0.05)	0.30*	(0.19; 0.41)	0.12	(−0.01; 0.25)	−0.28*	(−0.38; −0.18)	0.01	(−0.05; 0.07)	−0.27*	(−0.36; −0.17)
Between individual ICC	0.42		0.59		0.17		0.14		0.44		0.00	
**Area-level education**												
Low^†^												
Medium												
High												
**Area-level income**												
Low^†^												
Medium									−0.05	(−0.12; 0.02)		
High									0.05	(−0.03; 0.12)		
**Gini coefficient**												
Low^†^												
Medium	0.07*	(0.00; 0.14)										
High	0.01	(−0.06; 0.08)										
**Area Violence**												
Low^†^												
Medium			0.03	(−0.06; 0.12)			0.18*	(0.06; 0.30)				
High			−0.12*	(−0.21; −0.03)			0.09	(−0.03; 0.22)				
Between Area ICC	0.24		0.18		0.17		0.09		0.32		0.00	

yo: year-old, ICC: Intra-class Correlation.

^†^Reference category.

^*^p < 0.05.

Regarding contextual variables, in women, socio-economic inequality had a small effect on “irritable mood and headache” (β = 0.07), and residing in violent location was inversely associated with “chronic diseases and pain” (β = −0.12). For men, violence was associated with the pattern of “chronic pain and respiratory diseases” (β = 0.18). Furthermore, women with “chronic diseases and pain” and “substance use” presented little influence of area-level variables. While men with “chronic diseases” presented no influence of area-level variables, contextual influence in the occurrence of the pattern of “psychiatric disorders” among men was observed.

Regarding the use of health-care services, medical treatment was associated with “irritable mood and headache” in women (β = 0.36), and mental health-care was associated with “chronic diseases and pain” (β = 0.30). Among men, “chronic diseases” and “chronic pain and respiratory diseases” were associated with medical treatments (β = 0.33 and 0.25, respectively), and inversely associated with mental health-care (β = −0.27 and −0.28, respectively).

## Discussion

The presence of multiple conditions affecting the same individual is an important problem in the Brazilian population, even in younger brackets, and around one-fourth of adults aged 18–64 years-old presents a pattern of multimorbidity encompassing physical, mental illnesses or both. Additionally, men and women present distinct profiles of morbidity clustering, indicating the necessity of gender-focused preventive and intervention programs. To our knowledge, this is the first study, using a multilevel approach, which addresses the interrelationship of multiple conditions investigating physical but also mental disorders among younger adults of a general population. The strengths of the present work include the assessment of individuals of a developing country, with the combination of diagnosed psychiatric and physical illness in the classification of multimorbidity, and the examination of area-level determinants of disease clustering. The key findings of the present study have to be highlighted in the domain of public health.

In the present study, the prevalence of multimorbidity was twice higher than previously reported in Brazil^14^. An earlier study also suggested that multimorbidity likely affects older people, women, and those with low educational level^14^. In addition to these findings, we have shown that there was a salient sex difference, aging effect, and socioeconomic influence on the patterns of multimorbidity. Women and men presented different patterns of physical-mental multimorbidity^42^. While women were more affected by an irritable pattern of mood psychopathology and pain, men suffered more painful and respiratory diseases. However, the patterns of multimorbidity for both sexes could explain around 30% of the data variance and some low-prevalence diseases did not fit into any factor.

In comparison with previous findings, it rendered clear that the burden of multiple co-occurring chronic diseases is not high just in older age groups^13,43^, but a substantial proportion of economically-active younger adults in Brazil were also affected by multimorbidity. In the same direction, the Scottish study by Barnett and colleagues^13^ has indicated that the onset of multimorbidity might occur 10–15 years earlier in individuals living in deprived areas. Because chronic diseases generally start during peak economically-active years in LMICs^7^, multimorbidity clusters have disabling penalties and deeply affect the productivity of working populations, demanding prevention, early identification, and timely treatment.

Regarding the patterns of multimorbidity, despite heterogeneous methodology (number of selected diseases, diagnostic tool, and statistical procedures), three dimensions of multimorbidity were regularly identified^6^: cardiovascular and metabolic diseases; mental health problems; and musculoskeletal diseases. Previously, data from the Brazilian National Health Survey 2013^14^ had also reported an analogous pattern of multimorbidity. Our results were in line with these key findings: ‘irritable mood and headache’ was the most important pattern among women, while ‘chronic pain and respiratory diseases’ among men. Noteworthy, the individual- and area-level characteristics affect these clustering patterns.

The singular case of São Paulo, located in an upper-middle-income country in late epidemiological transition and with high rates of inequality, may anticipate the policy planning and gradual transformation of low-income countries toward an optimal functioning of patient-centred health systems. In LMICs, the inverse care law is persistently confirmed by the fact that highly deprived areas also present less availability of health-care services^44^. Moreover, the current Brazilian health-care system, which is highly focused in specialized clinics^21,22^, should be readapted and strengthened to accommodate the multimorbidity epidemic in a person-centred holistic approach. Therefore, the combined recognition of morbidities that frequently co-occur may reduce the need of multiple visits to overloaded professionals in LMICs, facilitating comprehensive management of clustered health-care needs in communities.

The example of São Paulo indicated that multimorbidity is dependent on contextual characteristics of residence, but the impact of improved living conditions on well-being and health indicators remains to be demonstrated. Lessons that have been learned from different LMICs should be used to reduce the vicious circle of adverse disadvantages^16,45–49^, with the integration and scaling-up of the mental health component in primary care, due to its close relationship with the physical component^50–52^. Forecasting the worldwide growth of the ageing population, policymakers will have to face multimorbidity as a global challenge in the near future. The current unmet needs of the general population^23–25^ already indicate that it is necessary to deliver earlier preventive and treatment programs by the health-care systems, especially in LMICs. Hence, preventive efforts should start well before older age, focusing those people living in areas of high socio-economic inequality^45^.

A further consequence of these findings refers to the education of the teams that will face the tasks of prevention and treatment of at-risk populations. The requirement of a multiple-disease approach needs to be strengthened in the medical training in order to fulfil the requirements indispensable to deal with this uncovering epidemiologic profile^13^. The educational emphasis of health-care professionals should incorporate longitudinal clerkship that helps students to acquire a broad understanding of the emerging challenges of long-term multimorbidity conditions presented by aging adults^53^. For example, skills for handling multiple diseases should be developed through the gradual integration of general clinical reasoning within the patients’ undivided physical-mental needs^54,55^.

### Limitations

Despite the several strengths of this study, our findings should be interpreted bearing in mind some limitations. Because the present study aimed to investigate common morbidities among the general population aged 18–64-year-old in their household, those treatment-seeking, institutionalized and homeless people were not included in the sample. Also, non-affective psychosis and other severe mental disorders were not included in the analysis. Likewise, the association between suicidality and multimorbidity was not considered as a potential outcome of this sample^56^.

Moreover, although the cross-sectional nature of the results presented herein has shown the size, patterns, and determinants of multimorbidity burden, the underlying causal relationships between co-occurring chronic diseases and patients’ disabilities need to be demonstrated in a more solid basis. The joint evolutionary course of multiple disorders might be captured with refined probabilistic models^57^.

## Conclusion

Multimorbidity is the greatest global challenge of the 21^st^ century. It affects not just older patients in HICs, but also the general population in LMICs. International health systems face a sizable proportion of people younger than 65 years with multimorbidity, whose management must be personalized. The government of LMICs should reorganize and coordinate health delivery to integrate the whole needs of people with multimorbidity, through training of health professionals and expansion of primary care. The clinician should be sensitive to the patient’s sex because distinctive patterns of illnesses affect women and men. The recovery of the economic burden of multimorbidity, as lost economic productivity due to multiple illnesses and premature deaths, begins with the recognition of key factors involved in its onset. Also, stakeholders must tailor structural financing for improving the living conditions of the disadvantaged people residing in deprived areas.

Future research priorities include uniform definition and measurement of multimorbidity to allow generating information with internal and external validity. The overlooked relationship between multimorbidity and suicidality should be explored in further studies^56^. Because increasing provision of treatment would not suffice to diminish the prevalence of common physical-mental morbidities in the general population^58,59^, balanced management of comorbid conditions in primary care would play a vital role in the framework of health systems, by shifting from a single-disease approach to a multimorbid approach.

## Supplementary information

S1

## Data Availability

The full dataset supporting the findings of the present observational study can be obtained upon request to the authors.
