# Supplementary material for: Multilevel Analysis of the Patterns of Physical-Mental Multimorbidity in General Population of São Paulo Metropolitan Area, Brazil
Source: Sci Rep. 2019 Feb 20;9:2390. doi: 10.1038/s41598-019-39326-8 (PMC6382818; doi:10.1038/s41598-019-39326-8)
Supplement: Supplementary file 1 — S1 [file 41598_2019_39326_MOESM1_ESM.docx]

**Supplementary Information:**

**Title:** Multilevel Analysis of the Patterns of Physical-Mental Multimorbidity in General Population of São Paulo Metropolitan Area, Brazil

**Authors:** Yuan-Pang Wang^1,*^, Bruno P. Nunes^2^, Bruno M. Coêlho^1^, Geilson L. Santana^1^, Carla F. do Nascimento^3^, Maria Carmen Viana^4^, Isabela M. Benseñor^5^, Laura H. Andrade^1,†^, Alexandre D.P. Chiavegatto Filho^3,†^

^1^ Nucleo de Epidemiologia Psiquiatrica (LIM-23), Instituto de Psiquiatria, Hospital das Clinicas HC FMUSP, Faculdade de Medicina, Universidade de Sao Paulo, Sao Paulo, SP, BR.

^2^ Nursing Department, Federal University of Pelotas RS, BR.

^3^ Department of Epidemiology, School of Public Health, University of São Paulo, Sao Paulo SP, BR.

^4^ Department of Social Medicine, Federal University of Espírito Santo, Vitória ES, BR.

^5^ Center for Clinical and Epidemiological Research, Hospital Universitario HU USP, Universidade de Sao Paulo, Sao Paulo, SP, BR.

* corresponding author: gnap_inbox@hotmail.com

^†^ these authors contributed equally to this work

**Supplementary Table S1:** Models of multilevel analysis of patterns of multimorbidity in the general population of the São Paulo metropolitan area, by sex

**S1-A: Women**

|  | **Women’s Factor 1: Irritable mood & headache** | | | | | | | | | | | | | | |
| --- | --- | --- | --- | --- | --- | --- | --- | --- | --- | --- | --- | --- | --- | --- | --- |
|  | **Model 1** | |  | **Model 2** | |  | **Model 3** | |  | **Model 4** | |  | **Model 5** | |  |
|  | **β** | **(95% CI)** |  | **β** | **(95% CI)** |  | **β** | **(95% CI)** |  | **β** | **(95% CI)** |  | **β** | **(95% CI)** | |
| **Age bracket** |  |  |  |  |  |  |  |  |  |  |  |  |  |  | |
| 18-34 yo**^†^** |  |  |  |  |  |  |  |  |  |  |  |  |  |  | |
| 35-49 yo | 0.04 | (-0.02; 0.10) |  | 0.04 | (-0.02; 0.10) |  | 0.04 | (-0.02; 0.10) |  | 0.04 | (-0.02; 0.10) |  | 0.04 | (-0.02; 0.10) | |
| 50-64 yo | -0.07 | (-0.14; 0.01) |  | -0.07 | (-0.14; 0.01) |  | -0.06 | (-0.14; 0.01) |  | -0.07 | (-0.15; 0.00) |  | -0.06 | (-0.14; 0.01) | |
| **Marital status** |  |  |  |  |  |  |  |  |  |  |  |  |  |  | |
| Single**^†^** |  |  |  |  |  |  |  |  |  |  |  |  |  |  | |
| Married/Cohabiting | 0.02 | (-0.05; 0.10) |  | 0.02 | (-0.05; 0.10) |  | 0.02 | (-0.05; 0.09) |  | 0.02 | (-0.05; 0.10) |  | 0.02 | (-0.05; 0.10) | |
| Separated/Widowed/Divorced | -0.01 | (-0.10; 0.08) |  | -0.01 | (-0.10; 0.08) |  | -0.01 | (-0.10; 0.08) |  | 0.00 | (-0.09; 0.09) |  | -0.01 | (-0.10; 0.08) | |
| **Educational level** |  |  |  |  |  |  |  |  |  |  |  |  |  |  | |
| 1**^†^** |  |  |  |  |  |  |  |  |  |  |  |  |  |  | |
| 2 | 0.04 | (-0.03; 0.12) |  | 0.04 | (-0.03; 0.12) |  | 0.05 | (-0.03; 0.12) |  | 0.04 | (-0.03; 0.11) |  | 0.04 | (-0.03; 0.12) | |
| 3 | 0.02 | (-0.06; 0.09) |  | 0.02 | (-0.06; 0.09) |  | 0.02 | (-0.06; 0.09) |  | 0.01 | (-0.06; 0.09) |  | 0.02 | (-0.06; 0.09) | |
| 4 | -0.02 | (-0.10; 0.07) |  | -0.01 | (-0.10; 0.07) |  | -0.01 | (-0.10; 0.08) |  | -0.02 | (-0.11; 0.07) |  | -0.01 | (-0.10; 0.08) | |
| **Severity** |  |  |  |  |  |  |  |  |  |  |  |  |  |  | |
| 1 | 1.82* | (1.73; 1.90) |  | 1.82* | (1.73; 1.90) |  | 1.82* | (1.73; 1.90) |  | 1.82* | (1.73; 1.90) |  | 1.82* | (1.73; 1.90) | |
| 2 | 1.45* | (1.37; 1.54) |  | 1.45* | (1.37; 1.54) |  | 1.45* | (1.37; 1.54) |  | 1.45* | (1.37; 1.54) |  | 1.45* | (1.37; 1.54) | |
| 3 | 1.23* | (1.14; 1.32) |  | 1.23* | (1.14; 1.32) |  | 1.23* | (1.14; 1.32) |  | 1.23* | (1.14; 1.32) |  | 1.23* | (1.14; 1.32) | |
| 4**^†^** |  |  |  |  |  |  |  |  |  |  |  |  |  |  | |
| **Medical treatment** |  |  |  |  |  |  |  |  |  |  |  |  |  |  | |
| No**^†^** |  |  |  |  |  |  |  |  |  |  |  |  |  |  | |
| Yes | 0.36* | (0.28; 0.44) |  | 0.36* | (0.28; 0.44) |  | 0.36* | (0.28; 0.44) |  | 0.36* | (0.28; 0.44) |  | 0.36* | (0.28; 0.44) | |
| **Mental health-care** |  |  |  |  |  |  |  |  |  |  |  |  |  |  | |
| No**^†^** |  |  |  |  |  |  |  |  |  |  |  |  |  |  | |
| Yes | -0.12* | (-0.21; -0.04) |  | -0.12* | (-0.21; -0.04) |  | -0.12* | (-0.21; -0.04) |  | -0.13* | (-0.21; -0.05) |  | -0.12* | (-0.21; -0.04) | |
| **Area-level education** |  |  |  |  |  |  |  |  |  |  |  |  |  |  | |
| Low**^†^** |  |  |  |  |  |  |  |  |  |  |  |  |  |  | |
| Medium |  |  |  | 0.03 | (-0.04; 0.10) |  |  |  |  |  |  |  |  |  | |
| High |  |  |  | 0.01 | (-0.07; 0.08) |  |  |  |  |  |  |  |  |  | |
| **Area-level income** |  |  |  |  |  |  |  |  |  |  |  |  |  |  | |
| Low**^†^** |  |  |  |  |  |  |  |  |  |  |  |  |  |  | |
| Medium |  |  |  |  |  |  | 0.02 | (-0.04; 0.09) |  |  |  |  |  |  | |
| High |  |  |  |  |  |  | -0.03 | (-0.10; 0.03) |  |  |  |  |  |  | |
| **Gini coefficient** |  |  |  |  |  |  |  |  |  |  |  |  |  |  | |
| Low**^†^** |  |  |  |  |  |  |  |  |  |  |  |  |  |  | |
| Medium |  |  |  |  |  |  |  |  |  | 0.07* | (0.00; 0.14) |  |  |  | |
| High |  |  |  |  |  |  |  |  |  | 0.01 | (-0.06; 0.08) |  |  |  | |
| **Area Violence** |  |  |  |  |  |  |  |  |  |  |  |  |  |  | |
| Low**^†^** |  |  |  |  |  |  |  |  |  |  |  |  |  |  | |
| Medium |  |  |  |  |  |  |  |  |  |  |  |  | -0.01 | (-0.08; 0.06) | |
| High |  |  |  |  |  |  |  |  |  |  |  |  | 0.03 | (-0.04; 0.10) | |
| **Intraclass Correlation (ICC)** | 0.41 |  |  | 0.49 |  |  | 0.33 |  |  | 0.24 |  |  | 0.44 |  | |

yo: year-old

**^†^** reference category

* *p* < 0.05

|  | **Women’s Factor 2: Chronic diseases & pain** | | | | | | | | | | | | | |  |
| --- | --- | --- | --- | --- | --- | --- | --- | --- | --- | --- | --- | --- | --- | --- | --- |
|  | **Model 1** | |  | **Model 2** | |  | **Model 3** | |  | **Model 4** | |  | **Model 5** | |  |
|  | **β** | **(95% CI)** |  | **β** | **(95% CI)** |  | **β** | **(95% CI)** |  | **β** | **(95% CI)** |  | **β** | **(95% CI)** | |
| **Age bracket** |  |  |  |  |  |  |  |  |  |  |  |  |  |  | |
| 18-34 yo**^†^** |  |  |  |  |  |  |  |  |  |  |  |  |  |  | |
| 35-49 yo | -0.31* | (-0.39; -0.23) |  | -0.31* | (-0.40; -0.23) |  | -0.31* | (-0.40; -0.23) |  | -0.31* | (-0.39; -0.23) |  | -0.31* | (-0.40; -0.23) | |
| 50-64 yo | -0.98* | (-1.09; -0.88) |  | -0.99* | (-1.10; -0.89) |  | -0.99* | (-1.09; -0.88) |  | -0.98* | (-1.09; -0.88) |  | -0.99* | (-1.10; -0.89) | |
| **Marital status** |  |  |  |  |  |  |  |  |  |  |  |  |  |  | |
| Single**^†^** |  |  |  |  |  |  |  |  |  |  |  |  |  |  | |
| Married/Cohabiting | -0.03 | (-0.13; 0.07) |  | -0.03 | (-0.13; 0.07) |  | -0.03 | (-0.13; 0.07) |  | -0.03 | (-0.13; 0.07) |  | -0.03 | (-0.13; 0.07) | |
| Separated/ Widowed/Divorced | 0.06 | (-0.06; 0.18) |  | 0.06 | (-0.06; 0.18) |  | 0.06 | (-0.06; 0.18) |  | 0.06 | (-0.06; 0.18) |  | 0.06 | (-0.06; 0.18) | |
| **Educational level** |  |  |  |  |  |  |  |  |  |  |  |  |  |  | |
| 1**^†^** |  |  |  |  |  |  |  |  |  |  |  |  |  |  | |
| 2 | 0.21* | (0.11; 0.32) |  | 0.21* | (0.11; 0.31) |  | 0.21* | (0.11; 0.31) |  | 0.21* | (0.11; 0.32) |  | 0.21* | (0.11; 0.31) | |
| 3 | 0.31* | (0.21; 0.42) |  | 0.31* | (0.20; 0.41) |  | 0.31* | (0.21; 0.41) |  | 0.31* | (0.21; 0.42) |  | 0.30* | (0.20; 0.40) | |
| 4 | 0.52* | (0.40; 0.64) |  | 0.50* | (0.38; 0.62) |  | 0.51* | (0.39; 0.63) |  | 0.52* | (0.40; 0.64) |  | 0.50* | (0.38; 0.63) | |
| **Severity** |  |  |  |  |  |  |  |  |  |  |  |  |  |  | |
| 1 | -0.28* | (-0.39; -0.16) |  | -0.28* | (-0.39; -0.16) |  | -0.28* | (-0.39; -0.16) |  | -0.28* | (-0.39; -0.16) |  | -0.28* | (-0.39; -0.16) | |
| 2 | -0.13* | (-0.24; -0.01) |  | -0.13* | (-0.24; -0.02) |  | -0.13* | (-0.24; -0.02) |  | -0.13* | (-0.24; -0.01) |  | -0.13* | (-0.24; -0.01) | |
| 3 | -0.09 | (-0.21; 0.04) |  | -0.09 | (-0.22; 0.03) |  | -0.09 | (-0.21; 0.03) |  | -0.09 | (-0.21; 0.04) |  | -0.09 | (-0.21; 0.04) | |
| 4**^†^** |  |  |  |  |  |  |  |  |  |  |  |  |  |  | |
| **Medical treatment** |  |  |  |  |  |  |  |  |  |  |  |  |  |  | |
| No**^†^** |  |  |  |  |  |  |  |  |  |  |  |  |  |  | |
| Yes | -0.21* | (-0.32; -0.10) |  | -0.21* | (-0.32; -0.10) |  | -0.21* | (-0.32; -0.10) |  | -0.21* | (-0.32; -0.10) |  | -0.21* | (-0.32; -0.10) | |
| **Mental health-care** |  |  |  |  |  |  |  |  |  |  |  |  |  |  | |
| No**^†^** |  |  |  |  |  |  |  |  |  |  |  |  |  |  | |
| Yes | 0.30* | (0.19; 0.41) |  | 0.30* | (0.19; 0.42) |  | 0.30* | (0.19; 0.41) |  | 0.30* | (0.19; 0.41) |  | 0.30* | (0.19; 0.41) | |
| **Area-level education** |  |  |  |  |  |  |  |  |  |  |  |  |  |  | |
| Low**^†^** |  |  |  |  |  |  |  |  |  |  |  |  |  |  | |
| Medium |  |  |  | 0.02 | (-0.08; 0.11) |  |  |  |  |  |  |  |  |  | |
| High |  |  |  | 0.09 | (-0.01; 0.19) |  |  |  |  |  |  |  |  |  | |
| **Area-level income** |  |  |  |  |  |  |  |  |  |  |  |  |  |  | |
| Low**^†^** |  |  |  |  |  |  |  |  |  |  |  |  |  |  | |
| Medium |  |  |  |  |  |  | -0.03 | (-0.12; 0.07) |  |  |  |  |  |  | |
| High |  |  |  |  |  |  | 0.07 | (-0.03; 0.16) |  |  |  |  |  |  | |
| **Gini coefficient** |  |  |  |  |  |  |  |  |  |  |  |  |  |  | |
| Low**^†^** |  |  |  |  |  |  |  |  |  |  |  |  |  |  | |
| Medium |  |  |  |  |  |  |  |  |  | 0.00 | (-0.10; 0.10) |  |  |  | |
| High |  |  |  |  |  |  |  |  |  | 0.00 | (-0.10; 0.10) |  |  |  | |
| **Area Violence** |  |  |  |  |  |  |  |  |  |  |  |  |  |  | |
| Low**^†^** |  |  |  |  |  |  |  |  |  |  |  |  |  |  | |
| Medium |  |  |  |  |  |  |  |  |  |  |  |  | 0.03 | (-0.06; 0.12) | |
| High |  |  |  |  |  |  |  |  |  |  |  |  | -0.12* | (-0.21; -0.03) | |
| **Intraclass Correlation (ICC)** | 0.59 |  |  | 0.48 |  |  | 0.43 |  |  | 0.69 |  |  | 0.18 |  | |

yo: year-old

**^†^** reference category

* *p* < 0.05

|  | **Women’s Factor 3: Substance use disorders** | | | | | | | | | | | | | |
| --- | --- | --- | --- | --- | --- | --- | --- | --- | --- | --- | --- | --- | --- | --- |
|  | **Model 1** | |  | **Model 2** | |  | **Model 3** | |  | **Model 4** | |  | **Model 5** | |
|  | **β** | **(95% CI)** |  | **β** | **(95% CI)** |  | **β** | **(95% CI)** |  | **β** | **(95% CI)** |  | **β** | **(95% CI)** |
| **Age bracket** |  |  |  |  |  |  |  |  |  |  |  |  |  |  |
| 18-34 yo * |  |  |  |  |  |  |  |  |  |  |  |  |  |  |
| 35-49 yo | -0.11* | (-0.21; -0.02) |  | -0.12* | (-0.21; -0.02) |  | -0.12* | (-0.21; -0.02) |  | -0.11* | (-0.21; -0.02) |  | -0.12* | (-0.21; -0.02) |
| 50-64 yo | -0.30* | (-0.42; -0.18) |  | -0.31* | (-0.42; -0.19) |  | -0.30* | (-0.42; -0.19) |  | -0.30* | (-0.42; -0.18) |  | -0.31* | (-0.43; -0.19) |
| **Marital status** |  |  |  |  |  |  |  |  |  |  |  |  |  |  |
| Single**^†^** |  |  |  |  |  |  |  |  |  |  |  |  |  |  |
| Married/Cohabiting | -0.05 | (-0.17; 0.06) |  | -0.05 | (-0.17; 0.06) |  | -0.05 | (-0.17; 0.06) |  | -0.05 | (-0.17; 0.06) |  | -0.05 | (-0.17; 0.06) |
| Separated/ Widowed/Divorced | 0.06 | (-0.07; 0.20) |  | 0.07 | (-0.07; 0.20) |  | 0.06 | (-0.07; 0.20) |  | 0.06 | (-0.07; 0.20) |  | 0.06 | (-0.07; 0.20) |
| **Educational level** |  |  |  |  |  |  |  |  |  |  |  |  |  |  |
| 1**^†^** |  |  |  |  |  |  |  |  |  |  |  |  |  |  |
| 2 | -0.07 | (-0.18; 0.05) |  | -0.07 | (-0.19; 0.04) |  | -0.07 | (-0.18; 0.05) |  | -0.07 | (-0.18; 0.05) |  | -0.07 | (-0.19; 0.04) |
| 3 | 0.01 | (-0.10; 0.13) |  | 0.01 | (-0.11; 0.12) |  | 0.01 | (-0.11; 0.13) |  | 0.01 | (-0.10; 0.13) |  | 0.00 | (-0.11; 0.12) |
| 4 | 0.02 | (-0.12; 0.16) |  | 0.01 | (-0.13; 0.15) |  | 0.01 | (-0.12; 0.15) |  | 0.02 | (-0.12; 0.16) |  | 0.00 | (-0.13; 0.14) |
| **Severity** |  |  |  |  |  |  |  |  |  |  |  |  |  |  |
| 1 | 0.42* | (0.29; 0.55) |  | 0.42* | (0.29; 0.55) |  | 0.42* | (0.29; 0.55) |  | 0.42* | (0.29; 0.55) |  | 0.42* | (0.29; 0.55) |
| 2 | 0.04 | (-0.09; 0.16) |  | 0.04 | (-0.09; 0.16) |  | 0.03 | (-0.09; 0.16) |  | 0.04 | (-0.09; 0.16) |  | 0.04 | (-0.09; 0.16) |
| 3 | 0.23* | (0.09; 0.37) |  | 0.22* | (0.08; 0.37) |  | 0.22* | (0.08; 0.37) |  | 0.23* | (0.09; 0.37) |  | 0.22* | (0.08; 0.36) |
| 4**^†^** |  |  |  |  |  |  |  |  |  |  |  |  |  |  |
| **Medical treatment** |  |  |  |  |  |  |  |  |  |  |  |  |  |  |
| No**^†^** |  |  |  |  |  |  |  |  |  |  |  |  |  |  |
| Yes | -0.22* | (-0.34; -0.09) |  | -0.22* | (-0.34; -0.09) |  | -0.22* | (-0.34; -0.09) |  | -0.22* | (-0.34; -0.09) |  | -0.22* | (-0.34; -0.09) |
| **Mental health-care** |  |  |  |  |  |  |  |  |  |  |  |  |  |  |
| No**^†^** |  |  |  |  |  |  |  |  |  |  |  |  |  |  |
| Yes | 0.12 | (-0.01; 0.25) |  | 0.12 | (-0.01; 0.25) |  | 0.12 | (-0.01; 0.24) |  | 0,12 | (-0.01; 0.25) |  | 0.12 | (-0.01; 0.24) |
| **Area-level education** |  |  |  |  |  |  |  |  |  |  |  |  |  |  |
| Low**^†^** |  |  |  |  |  |  |  |  |  |  |  |  |  |  |
| Medium |  |  |  | -0.01 | (-0.12; 0.09) |  |  |  |  |  |  |  |  |  |
| High |  |  |  | 0.03 | (-0.07; 0.13) |  |  |  |  |  |  |  |  |  |
| **Area-level income** |  |  |  |  |  |  |  |  |  |  |  |  |  |  |
| Low**^†^** |  |  |  |  |  |  |  |  |  |  |  |  |  |  |
| Medium |  |  |  |  |  |  | -0.04 | (-0.14; 0.07) |  |  |  |  |  |  |
| High |  |  |  |  |  |  | 0.02 | (-0.08; 0.13) |  |  |  |  |  |  |
| **Gini coefficient** |  |  |  |  |  |  |  |  |  |  |  |  |  |  |
| Low**^†^** |  |  |  |  |  |  |  |  |  |  |  |  |  |  |
| Medium |  |  |  |  |  |  |  |  |  | -0.02 | (-0.12; 0.08) |  |  |  |
| High |  |  |  |  |  |  |  |  |  | -0.01 | (-0.11; 0.09) |  |  |  |
| **Area Violence** |  |  |  |  |  |  |  |  |  |  |  |  |  |  |
| Low**^†^** |  |  |  |  |  |  |  |  |  |  |  |  |  |  |
| Medium |  |  |  |  |  |  |  |  |  |  |  |  | -0.01 | (-0.11; 0.09) |
| High |  |  |  |  |  |  |  |  |  |  |  |  | -0.08 | (-0.18; 0.02) |
| **Intraclass Correlation (ICC)** | 0.17 |  |  | 0.29 |  |  | 0.25 |  |  | 0.26 |  |  | 0.24 |  |

yo: year-old

**^†^** reference category

* *p* < 0.05

**S1-B: Men**

|  | **Men’s Factor 1: Chronic pain & respiratory disease** | | | | | | | | | | | | | |  |
| --- | --- | --- | --- | --- | --- | --- | --- | --- | --- | --- | --- | --- | --- | --- | --- |
|  | **Model 1** | |  | **Model 2** | |  | **Model 3** | |  | **Model 4** | |  | **Model 5** | | |
|  | **β** | **(95% CI)** |  | **β** | **(95% CI)** |  | **β** | **(95% CI)** |  | **β** | **(95% CI)** |  | **β** | **(95% CI)** |  |
| **Age bracket** |  |  |  |  |  |  |  |  |  |  |  |  |  |  |  |
| 18-34 yo**^†^** |  |  |  |  |  |  |  |  |  |  |  |  |  |  |  |
| 35-49 yo | 0.05 | (-0.06; 0.15) |  | 0.05 | (-0.06; 0.16) |  | 0.05 | (-0.06; 0.16) |  | 0.05 | (-0.06; 0.15) |  | 0.04 | (-0.06; 0.15) |  |
| 50-64 yo | 0.07 | (-0.06; 0.20) |  | 0.08 | (-0.05; 0.21) |  | 0.08 | (-0.05; 0.21) |  | 0.07 | (-0.06; 0.20) |  | 0.08 | (-0.05; 0.21) |  |
| **Marital status** |  |  |  |  |  |  |  |  |  |  |  |  |  |  |  |
| Single**^†^** |  |  |  |  |  |  |  |  |  |  |  |  |  |  |  |
| Married /Cohabiting | 0.19* | (0.07; 0.31) |  | 0.19* | (0.07; 0.31) |  | 0.19* | (0.07; 0.31) |  | 0.19* | (0.07; 0.31) |  | 0.19* | (0.07; 0.31) |  |
| Separated/ Widowed/Divorced | -0.04 | (-0.22; 0.13) |  | -0.04 | (-0.22; 0.14) |  | -0.04 | (-0.22; 0.14) |  | -0.04 | (-0.22; 0.14) |  | -0.04 | (-0.22; 0.14) |  |
| **Educational level** |  |  |  |  |  |  |  |  |  |  |  |  |  |  |  |
| 1**^†^** |  |  |  |  |  |  |  |  |  |  |  |  |  |  |  |
| 2 | -0.09 | (-0.22; 0.03) |  | -0.09 | (-0.22; 0.04) |  | -0.09 | (-0.22; 0.03) |  | -0.09 | (-0.22; 0.03) |  | -0.08 | (-0.21; 0.04) |  |
| 3 | -0.24* | (-0.36; -0.12) |  | -0.23* | (-0.36; -0.11) |  | -0.23* | (-0.36; -0.11) |  | -0.24* | (-0.36; -0.11) |  | -0.23* | (-0.35; -0.10) |  |
| 4 | -0.19* | (-0.34; -0.04) |  | -0.18* | (-0.33; -0.02) |  | -0.17* | (-0.32; -0.02) |  | -0.18* | (-0.33; -0.03) |  | -0.16* | (-0.31; -0.01) |  |
| **Severity** |  |  |  |  |  |  |  |  |  |  |  |  |  |  |  |
| 1 | 0.66* | (0.49; 0.83) |  | 0.66* | (0.49; 0.83) |  | 0.66* | (0.49; 0.83) |  | 0.66* | (0.49; 0.83) |  | 0.66* | (0.49; 0.83) |  |
| 2 | 0.59* | (0.40; 0.77) |  | 0.59* | (0.41; 0.77) |  | 0.59* | (0.41; 0.77) |  | 0.59* | (0.41; 0.77) |  | 0.59* | (0.41; 0.77) |  |
| 3 | 0.00 | (-0.16; 0.16) |  | 0.00 | (-0.16; 0.16) |  | 0.00 | (-0.16; 0.17) |  | 0.00 | (-0.16; 0.16) |  | -0.01 | (-0.17; 0.16) |  |
| 4**^†^** |  |  |  |  |  |  |  |  |  |  |  |  |  |  |  |
| **Medcial treatment** |  |  |  |  |  |  |  |  |  |  |  |  |  |  |  |
| No**^†^** |  |  |  |  |  |  |  |  |  |  |  |  |  |  |  |
| Yes | 0.25* | (0.07; 0.43) |  | 0.25* | (0.07; 0.43) |  | 0.25* | (0.06; 0.43) |  | 0.25* | (0.07; 0.43) |  | 0.25* | (0.06; 0.43) |  |
| **Mental health-care** |  |  |  |  |  |  |  |  |  |  |  |  |  |  |  |
| No**^†^** |  |  |  |  |  |  |  |  |  |  |  |  |  |  |  |
| Yes | -0.28* | (-0.38; -0.18) |  | -0.28* | (-0.38; -0.18) |  | -0.28* | (-0.38; -0.18) |  | -0.28* | (-0.38; -0.18) |  | -0.28* | (-0.38; -0.18) |  |
|  |  |  |  |  |  |  |  |  |  |  |  |  |  |  |  |
|  |  |  |  |  |  |  |  |  |  |  |  |  |  |  |  |
| **Area-level education** |  |  |  |  |  |  |  |  |  |  |  |  |  |  |  |
| Low**^†^** |  |  |  |  |  |  |  |  |  |  |  |  |  |  |  |
| Medium |  |  |  | -0.01 | (-0.15; 0.12) |  |  |  |  |  |  |  |  |  |  |
| High |  |  |  | -0.05 | (-0.19; 0.08) |  |  |  |  |  |  |  |  |  |  |
| **Area-level income** |  |  |  |  |  |  |  |  |  |  |  |  |  |  |  |
| Low**^†^** |  |  |  |  |  |  |  |  |  |  |  |  |  |  |  |
| Medium |  |  |  |  |  |  | -0.04 | (-0.17; 0.10) |  |  |  |  |  |  |  |
| High |  |  |  |  |  |  | -0.07 | (-0.20; 0.06) |  |  |  |  |  |  |  |
| **Gini coefficient** |  |  |  |  |  |  |  |  |  |  |  |  |  |  |  |
| Low**^†^** |  |  |  |  |  |  |  |  |  |  |  |  |  |  |  |
| Medium |  |  |  |  |  |  |  |  |  | -0.05 | (-0.18; 0.08) |  |  |  |  |
| High |  |  |  |  |  |  |  |  |  | 0.00 | (-0.14; 0.13) |  |  |  |  |
| **Area Violence** |  |  |  |  |  |  |  |  |  |  |  |  |  |  |  |
| Low**^†^** |  |  |  |  |  |  |  |  |  |  |  |  |  |  |  |
| Medium |  |  |  |  |  |  |  |  |  |  |  |  | 0.18* | (0.06; 0.30) |  |
| High |  |  |  |  |  |  |  |  |  |  |  |  | 0.09 | (-0.03; 0.22) |  |
| **Intraclass Correlation (ICC)** | 0.14 |  |  | 0.15 |  |  | 0.14 |  |  | 0.14 |  |  | 0.09 |  |  |

yo: year-old

**^†^** reference category

* *p* < 0.05

|  | **Men’s Factor 2: Psychiatric disorders** | | | | | | | | | | | | | |  |
| --- | --- | --- | --- | --- | --- | --- | --- | --- | --- | --- | --- | --- | --- | --- | --- |
|  | **Model 1** | |  | **Model 2** | |  | **Model 3** | |  | **Model 4** | |  | **Model 5** | |  |
|  | **β** | **(95% CI)** |  | **β** | **(95% CI)** |  | **β** | **(95% CI)** |  | **β** | **(95% CI)** |  | **β** | **(95% CI)** | |
| **Age bracket** |  |  |  |  |  |  |  |  |  |  |  |  |  |  | |
| 18-34 yo**^†^** |  |  |  |  |  |  |  |  |  |  |  |  |  |  | |
| 35-49 yo | -0.18* | (-0.25; -0.11) |  | -0.18* | (-0.25; -0.11) |  | -0.18* | (-0.25; -0.11) |  | -0.18* | (-0.25; -0.11) |  | -0.18* | (-0.25; -0.11) | |
| 50-64 yo | -0.30* | (-0.38; -0.22) |  | -0.31* | (-0.39; -0.22) |  | -0.30* | (-0.38; -0.22) |  | -0.30* | (-0.38; -0.22) |  | -0.30* | (-0.38; -0.22) | |
| **Marital status** |  |  |  |  |  |  |  |  |  |  |  |  |  |  | |
| Single**^†^** |  |  |  |  |  |  |  |  |  |  |  |  |  |  | |
| Married /Cohabiting | 0.00 | (-0.08; 0.07) |  | 0.00 | (-0.08; 0.07) |  | 0.00 | (-0.08; 0.07) |  | -0.01 | (-0.08; 0.07) |  | -0.01 | (-0.08; 0.07) | |
| Separated/ Widowed/Divorced | 0.02 | (-0.09; 0.13) |  | 0.02 | (-0.10; 0.13) |  | 0.02 | (-0.09; 0.13) |  | 0.02 | (-0.10; 0.13) |  | 0.02 | (-0.09; 0.13) | |
| **Educational level** |  |  |  |  |  |  |  |  |  |  |  |  |  |  | |
| 1**^†^** |  |  |  |  |  |  |  |  |  |  |  |  |  |  | |
| 2 | 0.03 | (-0.05; 0.11) |  | 0.02 | (-0.06; 0.10) |  | 0.02 | (-0.06; 0.10) |  | 0.03 | (-0.05; 0.11) |  | 0.02 | (-0.06; 0.10) | |
| 3 | 0.01 | (-0.07; 0.09) |  | 0.01 | (-0.07; 0.09) |  | 0.01 | (-0.07; 0.09) |  | 0.01 | (-0.07; 0.09) |  | 0.01 | (-0.07; 0.09) | |
| 4 | 0.01 | (-0.09; 0.10) |  | 0.00 | (-0.10; 0.10) |  | 0.00 | (-0.10; 0.09) |  | 0.01 | (-0.09; 0.10) |  | 0.00 | (-0.09; 0.10) | |
| **Severity** |  |  |  |  |  |  |  |  |  |  |  |  |  |  | |
| 1 | 2.37* | (2.27; 2.48) |  | 2.37* | (2.26; 2.48) |  | 2.37* | (2.26; 2.48) |  | 2.37* | (2.27; 2.48) |  | 2.37* | (2.26; 2.48) | |
| 2 | 1.67* | (1.56; 1.79) |  | 1.67* | (1.56; 1.79) |  | 1.67* | (1.56; 1.79) |  | 1.67* | (1.56; 1.79) |  | 1.67* | (1.56; 1.79) | |
| 3 | 1.57* | (1.46; 1.67) |  | 1.57* | (1.46; 1.67) |  | 1.57* | (1.47; 1.67) |  | 1.57* | (1.47; 1.67) |  | 1.57* | (1.47; 1.67) | |
| 4**^†^** |  |  |  |  |  |  |  |  |  |  |  |  |  |  | |
| **Medical treatment** |  |  |  |  |  |  |  |  |  |  |  |  |  |  | |
| No**^†^** |  |  |  |  |  |  |  |  |  |  |  |  |  |  | |
| Yes | 0.08 | (-0.04; 0.19) |  | 0.07 | (-0.04; 0.19) |  | 0.08 | (-0.04; 0.20) |  | 0.08 | (-0.04; 0.19) |  | 0.08 | (-0.04; 0.19) | |
| **Mental health-care** |  |  |  |  |  |  |  |  |  |  |  |  |  |  | |
| No**^†^** |  |  |  |  |  |  |  |  |  |  |  |  |  |  | |
| Yes | 0.01 | (-0.05; 0.07) |  | 0.01 | (-0.05; 0.07) |  | 0.01 | (-0.05; 0.07) |  | 0.01 | (-0.05; 0.07) |  | 0.01 | (-0.05; 0.07) | |
|  |  |  |  |  |  |  |  |  |  |  |  |  |  |  | |
| **Area-level education** |  |  |  |  |  |  |  |  |  |  |  |  |  |  | |
| Low**^†^** |  |  |  |  |  |  |  |  |  |  |  |  |  |  | |
| Medium |  |  |  | 0.04 | (-0.03; 0.11) |  |  |  |  |  |  |  |  |  | |
| High |  |  |  | 0.04 | (-0.03; 0.12) |  |  |  |  |  |  |  |  |  | |
| **Area-level income** |  |  |  |  |  |  |  |  |  |  |  |  |  |  | |
| Low**^†^** |  |  |  |  |  |  |  |  |  |  |  |  |  |  | |
| Medium |  |  |  |  |  |  | -0.05 | (-0.12; 0.02) |  |  |  |  |  |  | |
| High |  |  |  |  |  |  | 0.05 | (-0.03; 0.12) |  |  |  |  |  |  | |
| **Gini coefficient** |  |  |  |  |  |  |  |  |  |  |  |  |  |  | |
| Low**^†^** |  |  |  |  |  |  |  |  |  |  |  |  |  |  | |
| Medium |  |  |  |  |  |  |  |  |  | 0.03 | (-0.05; 0.10) |  |  |  | |
| High |  |  |  |  |  |  |  |  |  | -0.01 | (-0.08; 0.07) |  |  |  | |
| **Area Violence** |  |  |  |  |  |  |  |  |  |  |  |  |  |  | |
| Low**^†^** |  |  |  |  |  |  |  |  |  |  |  |  |  |  | |
| Medium |  |  |  |  |  |  |  |  |  |  |  |  | -0.03 | (-0.11; 0.04) | |
| High |  |  |  |  |  |  |  |  |  |  |  |  | -0.01 | (-0.08; 0.07) | |
| **Intraclass Correlation (ICC)** | 0.44 |  |  | 0.54 |  |  | 0.32 |  |  | 0.58 |  |  | 0.53 |  | |

yo: year-old

**^†^** reference category

* *p* < 0.05

|  | **Men’s Factor 3: Chronic diseases** | | | | | | | | | | | | | |
| --- | --- | --- | --- | --- | --- | --- | --- | --- | --- | --- | --- | --- | --- | --- |
|  | **Model 1** | |  | **Model 2** | |  | **Model 3** | |  | **Model 4** | |  | **Model 5** | |
|  | **β** | **(95% CI)** |  | **β** | **(95% CI)** |  | **β** | **(95% CI)** |  | **β** | **(95% CI)** |  | **β** | **(95% CI)** |
| **Age bracket** |  |  |  |  |  |  |  |  |  |  |  |  |  |  |
| 18-34 yo **^†^** |  |  |  |  |  |  |  |  |  |  |  |  |  |  |
| 35-49 yo | 0.30* | (0.20; 0.41) |  | 0.30* | (0.20; 0.41) |  | 0.31* | (0.20; 0.41) |  | 0.31* | (0.20; 0.41) |  | 0.30* | (0.20; 0.41) |
| 50-64 yo | 0.83* | (0.71; 0.95) |  | 0.83* | (0.71; 0.96) |  | 0.84* | (0.72; 0.96) |  | 0.84* | (0.71; 0.96) |  | 0.83* | (0.71; 0.95) |
| **Marital status** |  |  |  |  |  |  |  |  |  |  |  |  |  |  |
| Single**^†^** |  |  |  |  |  |  |  |  |  |  |  |  |  |  |
| Married/Cohabiting | 0.05 | (-0.06; 0.16) |  | 0.05 | (-0.07; 0.16) |  | 0.05 | (-0.07; 0.16) |  | 0.05 | (-0.07; 0.16) |  | 0.05 | (-0.06; 0.16) |
| Separated/ Widowed/Divorced | 0.10 | (-0.07; 0.26) |  | 0.10 | (-0.07; 0.26) |  | 0.10 | (-0.07; 0.27) |  | 0.10 | (-0.07; 0.26) |  | 0.10 | (-0.07; 0.26) |
| **Educational level** |  |  |  |  |  |  |  |  |  |  |  |  |  |  |
| 1**^†^** |  |  |  |  |  |  |  |  |  |  |  |  |  |  |
| 2 | -0.05 | (-0.17; 0.06) |  | -0.05 | (-0.17; 0.06) |  | -0.05 | (-0.17; 0.07) |  | -0.05 | (-0.17; 0.06) |  | -0.06 | (-0.17; 0.06) |
| 3 | -0.09 | (-0.20; 0.03) |  | -0.09 | (-0.20; 0.03) |  | -0.08 | (-0.20; 0.03) |  | -0.08 | (-0.20; 0.03) |  | -0.09 | (-0.20; 0.03) |
| 4 | -0.13 | (-0.27; 0.00) |  | -0.13 | (-0.27; 0.01) |  | -0.11 | (-0.25; 0.03) |  | -0.12 | (-0.26; 0.02) |  | -0.14 | (-0.28; 0.00) |
| **Severity** |  |  |  |  |  |  |  |  |  |  |  |  |  |  |
| 1 | 0.36* | (0.20; 0.52) |  | 0.36* | (0.20; 0.52) |  | 0.36* | (0.20; 0.52) |  | 0.36* | (0.20; 0.52) |  | 0.35* | (0.19; 0.51) |
| 2 | 0.75* | (0.58; 0.92) |  | 0.75* | (0.58; 0.92) |  | 0.75* | (0.58; 0.92) |  | 0.75* | (0.58; 0.92) |  | 0.75* | (0.58; 0.92) |
| 3 | 0.30* | (0.15; 0.45) |  | 0.30* | (0.15; 0.45) |  | 0.30* | (0.15; 0.45) |  | 0.30* | (0.15; 0.45) |  | 0.30* | (0.15; 0.45) |
| 4**^†^** |  |  |  |  |  |  |  |  |  |  |  |  |  |  |
| **Medical treatment** |  |  |  |  |  |  |  |  |  |  |  |  |  |  |
| No**^†^** |  |  |  |  |  |  |  |  |  |  |  |  |  |  |
| Yes | 0.33* | (0.16; 0.50) |  | 0.33* | (0.16; 0.50) |  | 0.33* | (0.16; 0.50) |  | 0.33* | (0.16; 0.50) |  | 0.33* | (0.16; 0.50) |
| **Mental health-care** |  |  |  |  |  |  |  |  |  |  |  |  |  |  |
| No**^†^** |  |  |  |  |  |  |  |  |  |  |  |  |  |  |
| Yes | -0.27* | (-0.36; -0.17) |  | -0.27* | (-0.36; -0.17) |  | -0.27* | (-0.36; -0.17) |  | -0.27* | (-0.36; -0.17) |  | -0.27* | (-0.36; -0.17) |
|  |  |  |  |  |  |  |  |  |  |  |  |  |  |  |
|  |  |  |  |  |  |  |  |  |  |  |  |  |  |  |
| **Area-level education** |  |  |  |  |  |  |  |  |  |  |  |  |  |  |
| Low**^†^** |  |  |  |  |  |  |  |  |  |  |  |  |  |  |
| Medium |  |  |  | -0.01 | (-0.11; 0.08) |  |  |  |  |  |  |  |  |  |
| High |  |  |  | -0.02 | (-0.11; 0.08) |  |  |  |  |  |  |  |  |  |
| **Area-level income** |  |  |  |  |  |  |  |  |  |  |  |  |  |  |
| Low**^†^** |  |  |  |  |  |  |  |  |  |  |  |  |  |  |
| Medium |  |  |  |  |  |  | -0.04 | (-0.14; 0.06) |  |  |  |  |  |  |
| High |  |  |  |  |  |  | -0.07 | (-0.17; 0.03) |  |  |  |  |  |  |
| **Gini coefficient** |  |  |  |  |  |  |  |  |  |  |  |  |  |  |
| Low**^†^** |  |  |  |  |  |  |  |  |  |  |  |  |  |  |
| Medium |  |  |  |  |  |  |  |  |  | -0.03 | (-0.13; 0.07) |  |  |  |
| High |  |  |  |  |  |  |  |  |  | -0.06 | (-0.16; 0.04) |  |  |  |
| **Area Violence** |  |  |  |  |  |  |  |  |  |  |  |  |  |  |
| Low**^†^** |  |  |  |  |  |  |  |  |  |  |  |  |  |  |
| Medium |  |  |  |  |  |  |  |  |  |  |  |  | -0.02 | (-0.12; 0.07) |
| High |  |  |  |  |  |  |  |  |  |  |  |  | -0.01 | (-0.11; 0.09) |
| **Intraclass Correlation (ICC)** | 0.00 |  |  | 0.00 |  |  | 0.00 |  |  | 0.00 |  |  | 0.00 |  |

yo: year-old

**^†^** reference category

* *p* < 0.05
